# XFEL diffraction: developing processing methods to optimize data quality

**DOI:** 10.1107/S1600577514028203

**Published:** 2015-01-29

**Authors:** Nicholas K. Sauter

**Affiliations:** a Physical Biosciences Division, Lawrence Berkeley National Laboratory, Berkeley, CA 94720, USA

**Keywords:** serial femtosecond crystallography, X-ray free-electron laser, partiality, postrefinement, mosaicity

## Abstract

Serial crystallography, using either femtosecond X-ray pulses from free-electron laser sources or short synchrotron-radiation exposures, has the potential to reveal metalloprotein structural details while minimizing damage processes. However, deriving a self-consistent set of Bragg intensities from numerous still-crystal exposures remains a difficult problem, with optimal protocols likely to be quite different from those well established for rotation photography. Here several data processing issues unique to serial crystallography are examined. It is found that the limiting resolution differs for each shot, an effect that is likely to be due to both the sample heterogeneity and pulse-to-pulse variation in experimental conditions. Shots with lower resolution limits produce lower-quality models for predicting Bragg spot positions during the integration step. Also, still shots by their nature record only partial measurements of the Bragg intensity. An approximate model that corrects to the full-spot equivalent (with the simplifying assumption that the X-rays are monochromatic) brings the distribution of intensities closer to that expected from an ideal crystal, and improves the sharpness of anomalous difference Fourier peaks indicating metal positions.

## Abstract 2

Bragg spots recorded from a still crystal necessarily give partial measurements of the structure factor intensity. Correction to the full-spot equivalent, relying on both a physical model for crystal disorder and postrefinement of the crystal orientation, improves the electron density map in serial crystallography.

## 1. Introduction   

As a strategy to avoid radiation damage, serial crystallography techniques aim to spread the X-ray dose over numerous crystal specimens, with the goal of observing Bragg spots from material that is close to the undamaged state. Based on the general decay of diffraction at a third-generation synchrotron source, an upper limit for radiation absorbed dose of 30 MGy has been proposed (Owen *et al.*, 2006 ▶) for single-crystal experiments. However, it is also clear that, even at doses far below this limit, damage at specific sites of interest is observed, in particular at metal sites where valence states and coordination geometry are sensitive to X-rays. In photosystem II (PSII), for example, the valence state of the multinuclear Mn_4_Ca complex can be monitored by X-ray absorption near-edge spectroscopy (XANES; Yano *et al.*, 2005 ▶). This complex, which is responsible for catalyzing the water oxidation reaction that evolves oxygen during photosynthesis, has a high valent Mn_4_(III_2_, IV_2_) structure in dark-adapted crystals. Critically, XANES can detect the accumulation of the radiation-damaged low-valent Mn(II) state even at the smallest doses examined, 0.6 MGy (at 100 K, with 13.3 keV radiation; Yano *et al.*, 2005 ▶). In contrast, the femtosecond-scale pulse durations from an X-ray free-electron laser (XFEL) permit the observation of the undamaged Mn_4_(III_2_, IV_2_) complex (Kern *et al.*, 2012 ▶, 2013 ▶), as shown by Mn *K*β_1,3_ X-ray emission spectroscopy that is likewise sensitive to the valence state (Alonso-Mori *et al.*, 2012 ▶). Short pulses also permit the direct observation of metal coordination bond lengths expected by theory from undamaged Mn (Suga *et al.*, 2015 ▶). Furthermore, these XFEL-based observations can be performed under room-temperature conditions that permit time-dependent pump–probe studies of the water oxidation mechanism (Kern *et al.*, 2014 ▶). Such experiments, when performed at the Linac Coherent Light Source (LCLS) with typical pulse durations of 40 fs, deliver a photon flux that would be equivalent to about 200 MGy (Kern *et al.*, 2012 ▶) if they were carried out at synchrotron-source time scales that allow radiation absorption. Thus, despite reports of diffraction decay with exceedingly long XFEL pulses (150 fs) and higher equivalent doses of 3 GGy (Lomb *et al.*, 2011 ▶), it appears that short-pulse XFEL still shots provide a promising method to look at radiation-sensitive structures, including those of metalloproteins.

Several high-resolution crystal structures have now been derived from XFEL diffraction (Boutet *et al.*, 2012 ▶; Redecke *et al.*, 2013 ▶; Barends *et al.*, 2013*a* ▶,*b* ▶; Liu *et al.*, 2013 ▶; Weierstall *et al.*, 2014 ▶; Hattne *et al.*, 2014 ▶; Kern *et al.*, 2014 ▶; Sawaya *et al.*, 2014 ▶; Cohen *et al.*, 2014 ▶; Tenboer *et al.*, 2014 ▶), with a common result being the large number of diffraction images required to produce a complete set of merged structure factors, ranging in these cases from 10^4^ to 1.8 × 10^5^. Part of this requirement arises from the heterogeneous quality of the diffraction images. When the data are examined in detail, it appears that the limiting resolution differs from shot to shot; this can be quantified by asking at what resolution the average Bragg spot signal-to-noise ratio [*I*/σ(*I*), where *I* is the intensity and 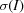 is the standard deviation from counting statistics] falls below a threshold value (Kern *et al.*, 2012 ▶, 2014 ▶; Hattne *et al.*, 2014 ▶). Scoring the data in this way suggests that only a small fraction of the images contributes signal at the outer limits of resolution. Considering this, the high resolution data are especially valuable, and special effort is warranted to optimize their measurement.

However, there are several known issues with XFEL data processing that make it difficult to gain accurate measurements of the high resolution signal. Firstly, there are tradeoffs made when implementing the algorithm for Bragg spot integration. The program *cctbx.xfel* (Hattne *et al.*, 2014 ▶) chooses small integration masks that tightly conform to the pixels believed to contain signal based on a lattice model, so as to discard surrounding pixels that contain only Gaussian noise. However, these small integration masks make the model highly sensitive to the calibration of both the detector distance and the detector metrology, which defines the mutual positions of sensor tiles (Hart *et al.*, 2012 ▶; Hattne *et al.*, 2014 ▶). While *cctbx.xfel* can fortunately calibrate sensor positions to about 0.1-pixel accuracy, it is found that even a 0.5-pixel miscalibration noticeably degrades the integrated data, and that this is felt most acutely for the highest-resolution data (Hattne *et al.*, 2014 ▶). Secondly, when performing a control data analysis with simulated image data, it can be shown that the inability to perfectly model the lattice orientation produces some Bragg spot predictions that are not in the simulated data, and misses other spots that really are in the simulation (Sauter *et al.*, 2014 ▶). These effects are most pronounced at the highest resolution limits. Thirdly, correct modeling of the diffraction pattern of a crystal with mosaic structure (Nave, 1998 ▶, 2014 ▶) requires counterbalancing parameters that describe the crystal’s physical properties. Increasing the parameter describing the angular spread of mosaic blocks permits the modeling of Bragg spots at the highest resolution limits, while decreasing the mosaic block size parameter allows the model to cover the low resolution Bragg spots. However, tuning these parameters requires assumptions about the mosaic structure of the crystal, and this entails increased uncertainty at the resolution extremes. Finally, in contrast to the usual rotation method employed at synchrotron sources, where each reciprocal lattice point is fully moved through the reflection condition, Bragg spots recorded on still shots necessarily represent partial measurements of the structure factor intensity. While it has been shown experimentally that adjacent spots can have differing partialities of measurement (Hattne *et al.*, 2014 ▶), particularly at high resolution, there is as yet no robust model to correct measurements to the full-spot equivalent.

Here further evidence that the data quality is most sensitive to error at the highest resolution is presented, providing further incentive for resolution-based filtering. However, it is demonstrated that a straightforward filter based on *I*/σ(*I*) removes real signal that is capable of improving anomalous difference measurements. Finally, with the eventual goal of deriving a proper expression to correct for partiality, it is demonstrated that a simplified model based on the assumption of monochromaticity provides a reasonable first step toward improving the structure factors.

## 2. Methods   

Data were processed with the program *cctbx.xfel* (Hattne *et al.*, 2014 ▶; Sauter *et al.*, 2014 ▶). A tutorial for processing the thermolysin data is presented at http://cci.lbl.gov/xfel.

### 2.1. Data analyzed   

Thermolysin diffraction patterns were reprocessed from a previously described data set (Hattne *et al.*, 2014 ▶) that is publicly archived at the Coherent X-ray Imaging Data Bank (http://cxidb.org), accession ID 23. Data were acquired during the L498 (December 2012) beam time at the 1 µm focus of the Coherent X-ray Imaging (CXI) instrument (Boutet & Williams, 2010 ▶) of LCLS. Typical crystal size was approximately 2 µm × 3 µm × 1 µm (Sierra *et al.*, 2012 ▶). Since the thermolysin structure contains a single Zn atom, it was possible to use the signal-to-noise ratio of the anomalous difference electron density as a metric for the data processing quality (Sauter *et al.*, 2014 ▶). Therefore, in the work presented here the analysis was limited to data (runs 16–27) collected at a wavelength of 1.269 Å, slightly more energetic than the Zn *K*-edge at 1.284 Å.

Simulated still-shot diffraction patterns from photosystem I (PSI) were obtained from James Holton (LBNL), and are available at http://bl831a.als.lbl.gov/example_data_sets/Illuin/LCLS. The 20000-image simulated dataset was created with the program *fastBragg* as described (Kirian *et al.*, 2010 ▶, 2011 ▶), utilizing modeled structure factors from Protein Data Bank entry 1jb0. Spatially coherent simulations of randomly oriented parallelepiped nanocrystals (17 × 17 × 30 unit cells; cell lengths *a* = *b* = 281 Å, *c* = 165.2 Å) were performed, assuming constant-flux, polarized, monochromatic radiation (λ = 1.32 Å) with zero divergence, impinging on a pixel-array detector with pixel size (0.11 mm)^2^ at a distance of 129 mm from the sample. Solvent scattering and shot noise were added so as to effectively limit the resolution to about 3.3 Å. At very low resolutions (*d* > 60 Å) the simulation exhibits diffraction fringes between Bragg spots as previously observed for PSI (Chapman *et al.*, 2011 ▶); however, the present paper attempts to analyze only the central Bragg peak, and the analysis is limited to the 15–3.5 Å range. Angular misorientation between the *cctbx.xfel* models and the true crystal orientations used for the simulation were calculated after accounting for the orientational ambiguities due to the hexagonal lattice symmetry operators (six-fold along **c** and two-fold along **a** + **b**). Angular misorientations were then decomposed into a rotation *R*_*z*_ about an axis parallel to the beam, and a residual rotation *R*_*xy*_ about an axis perpendicular to the beam.

### 2.2. Correction of the integrated intensity to the full-spot equivalent   

**Figure 1:**
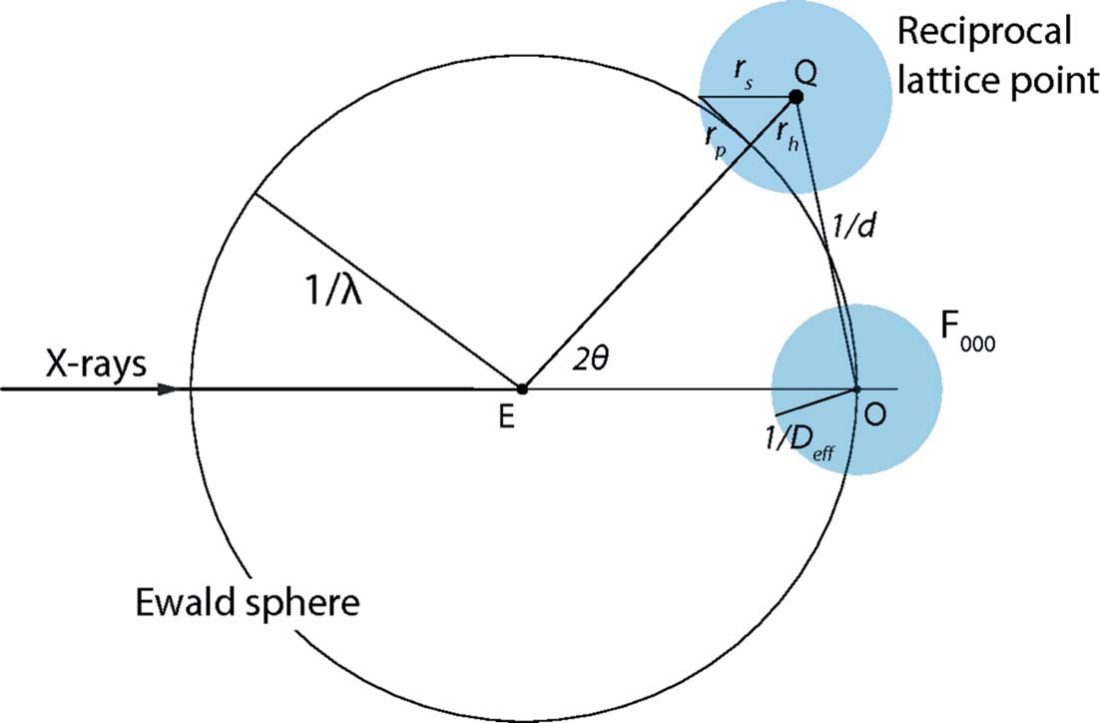
Geometric definition of partiality, accounting for the mosaic structure of the crystal. For a still shot taken with monochromatic X-rays of wavelength λ, a reciprocal lattice point (blue ball centered on *Q*) partially intersects the Ewald sphere. The intersection area, which is actually a spherical cap, is approximated by a circle of radius 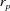, which is determined by 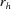, the distance from *Q* to the Ewald sphere, and 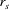, the resolution-dependent radius of the reciprocal lattice point as described in the text. Partiality is defined as the intersection area-to-ball volume ratio for lattice point *Q*, normalized by the intersection area-to-ball volume ratio of the *F*_000_ spot at reciprocal space origin *O*.

This section describes the component of partiality that arises from the crystal’s mosaic structure (Nave, 1998 ▶, 2014 ▶), setting aside the effects of beam properties such as dispersion and divergence for future work. Consider a reciprocal lattice point at reciprocal space position *Q* (rlpQ; Fig. 1 ▶). Points on the Ewald sphere of radius 1/λ (λ, wavelength) satisfy the reflection conditions exactly, but what if rlpQ is located a distance 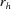 from the Ewald sphere surface, as in Fig. 1 ▶? In this case diffraction can still be modeled if crystal imperfections are taken into account, thus widening rlpQ into a ball of radius 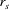, and satisfying the Laue conditions at the spherical cap-shaped intersection between the Ewald sphere and the rlpQ ball. Although this intersection area 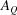 could be expressed analytically, it is convenient to approximate it as a circle of radius 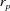 given by the right triangle in Fig. 1 ▶, with
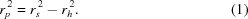
To obtain the best match with experimentally observed still-shot diffraction (Sauter *et al.*, 2014 ▶), it is useful to consider two parameters that contribute to the ball radius 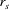. Viewing the crystal as a mosaic of coherently scattering blocks (Nave, 1998 ▶) of effective width 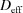 gives
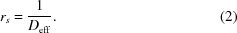
Meanwhile, considering the angular spread and unit cell variation among the mosaic blocks leads to the expression
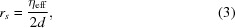
where *d* is the resolution and 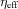 is the effective full-width mosaicity.^1^ Combining these two effects gives a final expression for the Ewald sphere intersection area:
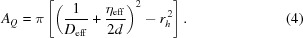
For the partiality of the Bragg spot in Fig. 1 ▶, arising from the crystal’s mosaic structure, it seems intuitive that the partiality should be proportional to 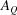, with a maximum obtained when the Ewald sphere slices through the center of rlpQ, and a minimum of 0 at 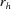 = 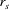. Taking the simplest case first, that with 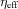 = 0, one finds that the maximal area is a constant: 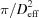. To turn this into a measure for partiality, one must assure that the partiality always takes on values from 0 to 1, and that it is a unitless quantity instead of having dimensions of length^−2^. This is accomplished by taking a suggestion from James Holton who, considering work on NaCl where a reference reflection was used (Bragg *et al.*, 1921 ▶), proposed (private communication) that the ratio between 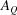 and the area intersected by the *F*_000_ reciprocal lattice point should be used:
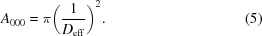
For *F*_000_, equation (5) always holds because 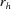 and 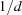 are identically zero.

**Figure 2:**
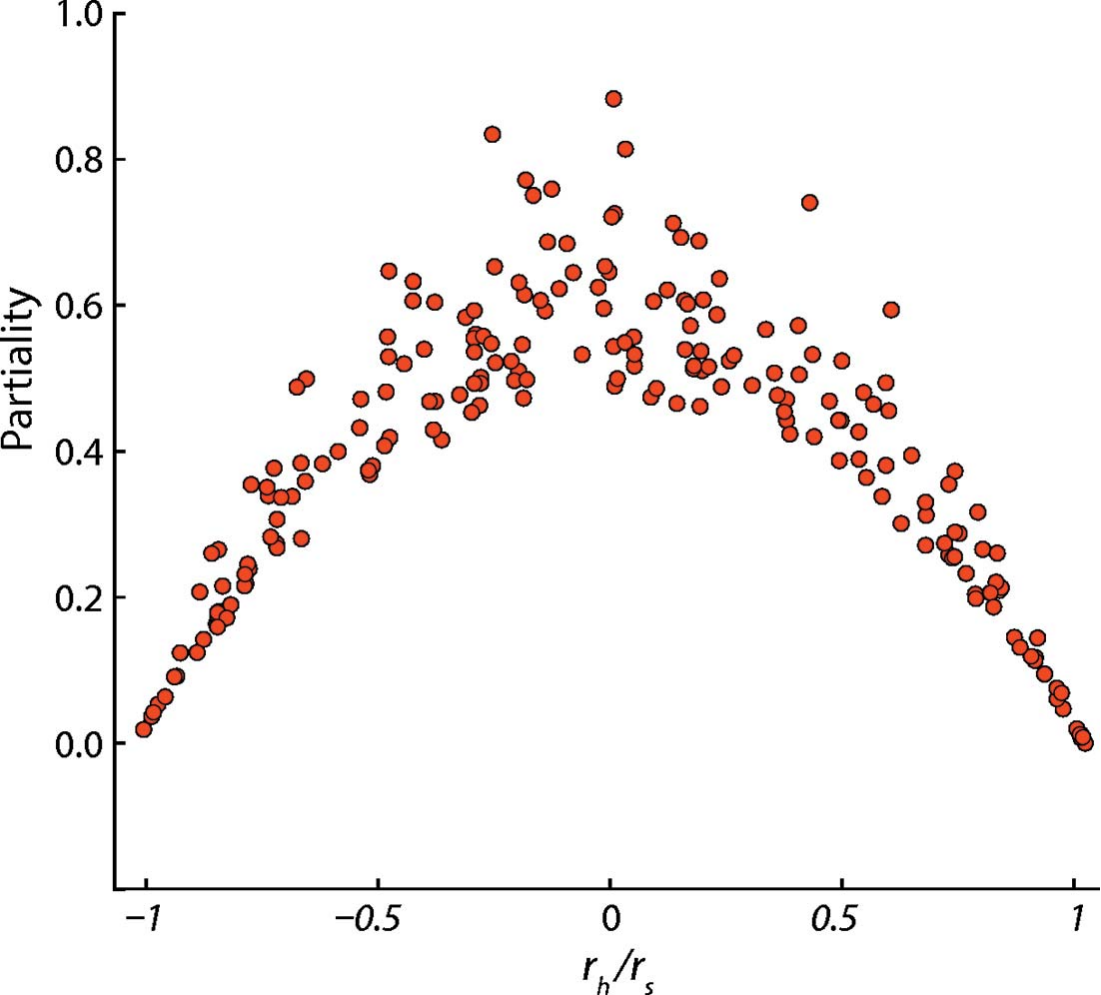
Partiality estimates for Bragg spots integrated on a single thermolysin image, plotted as a function of the 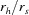 ratio.

Next, in the general case where 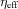 > 0, the maximal intersection area 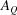 increases as a function of resolution due to its dependence on 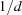, but this apparently goes against the expectation that the maximum partiality for any spot, independent of resolution, should be 1. To correct for this, one can normalize against the volume *V*_*Q*_ of the reciprocal lattice point, so that the full expression for partiality *P* involves the ratio of area to volume:
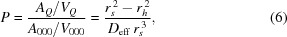
where
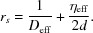
To evaluate equation (6), the parameters 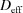 and 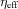 are determined separately for each image as previously described (Sauter *et al.*, 2014 ▶). Plotting the partiality of Bragg spots from a single thermolysin image (Fig. 2 ▶) confirms the expected behavior: the distribution of *P* increases to a maximum at 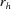 = 0 but never actually reaches 1.0 due to the normalization by 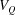, and it falls off to zero at 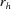 = 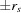.

Individual Bragg spot intensity measurements *I* are corrected to their full-spot equivalent 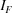 with
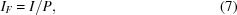
and those measurements with very low partiality are discarded, *i.e.* those with 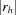 > 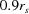.

Prior to merging data from different images together, duplicate measurements from different images are placed on a common scale by determining a separate scaling factor *G* and isotropic temperature factor *B* for each image. In common with previous work on scaling (Hamilton *et al.*, 1965 ▶; Fox & Holmes, 1966 ▶; Bolotovsky *et al.*, 1998 ▶; Kabsch, 2014 ▶), here these parameters were determined by iterative non-linear least-squares minimization of a target functional,
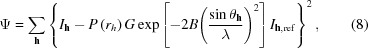
where 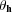 is the Bragg angle, and the summation is over all Miller indices 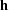 measured on a given image. However, instead of taking 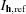 to be the best least-squares estimate of the structure factor intensity over the global dataset, the shortcut is taken of using reference intensities measured at a synchrotron, in this case thermolysin intensities from Protein Data Bank entry 2tli.^2^

Furthermore, for the computation of partiality in still-shot data [equation (6)], the Ewald sphere distances 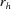 are sensitive functions of the crystal orientation, and in particular are susceptible to rotational uncertainties about the two orthogonal axes perpendicular to the X-ray beam; see §3.3 below. By expressing 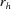 explicitly as a function of these rotations, the scaling equation (8) can be modified to include these rotations as free parameters. The necessary equation is:
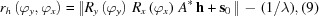
where 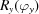 and 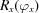 are matrices describing rotational perturbations through angles 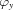 and 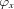 about orthogonal axes *y* and *x* perpendicular to the beam, 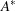 is the reciprocal space orientation matrix determined by indexing (Sauter *et al.*, 2006 ▶), also known as the *UB* matrix (Busing & Levy, 1967 ▶), and 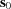 is the vector describing the travel direction of the X-ray beam with length 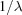. With the final set of free parameters being *G*, *B*, 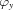 and 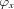, this adjustment of the crystal orientation to optimize agreement between reference intensities and the corrected measured intensities is similar to other post­refinement protocols used for both classical rotation photography (Winkler *et al.*, 1979 ▶; Rossmann *et al.*, 1979 ▶) and XFEL scaling (White, 2014 ▶; Kabsch, 2014 ▶).

### 2.3. Comparison of data processing protocols   

The thermolysin data were processed five times to assess the relative effects of differing protocols (Table 1 ▶). During the integration step, the lattice models were either truncated (Hattne *et al.*, 2014 ▶) at a resolution limit, separate for each lattice, where integrated intensity measurements fell below a threshold value (protocols 4, 6 and 7POST); or the data were integrated to a fixed limit of 2.2 Å (6F and 7F,POST). In either case, negative measurements were removed before the data from separate lattices were scaled together. Scaling was performed either by finding a simple scaling constant to fit partial structure factor intensities from each lattice to full calculated intensities based on PDB entry 2tli (4, 6 and 6F) as previously described (Hattne *et al.*, 2014 ▶), or by the post­refinement protocol of §2.2 (7POST and 7F,POST). Once duplicate measurements were merged globally over the whole data set, intensity distribution statistics were calculated with *phenix.xtriage* (Zwart *et al.*, 2005 ▶). The previously published XFEL thermolysin structure (PDB entry 4ow3) was re-refined against the newly processed data with *phenix.refine* (Afonine *et al.*, 2012 ▶), and anomalous difference Fourier peak heights analyzed with *phenix* (Adams *et al.*, 2010 ▶). Likelihood-weighted maps displayed with *Coot* (Emsley *et al.*, 2010 ▶) are shown in Fig. S1.^3^ Correlation coefficients of these maps to a 1.65 Å reference model (from synchrotron-based data, PDB entry 2tlx) were determined after rigid body refinement of the 2tlx model into the XFEL unit cell. Separately, in order to assess the ability to perform automated model building, the structure was solved by molecular replacement against 4ow3 with *phaser* (McCoy *et al.*, 2007 ▶). Molecular replacement phasing information was combined with single-wavelength anomalous differences with *phenix.autosol* (Terwilliger *et al.*, 2009 ▶), and fully automated fitting of the amino acid sequence was performed with *phenix.autobuild* (Terwilliger *et al.*, 2008 ▶).

## 3. Results and discussion   

### 3.1. Shot-to-shot heterogeneity is intrinsic to the data   

Heterogeneity in XFEL-based serial crystallographic images is a necessary consequence of physical properties such as the mosaic structure that varies among crystals, and pulse-to-pulse differences in incident flux and the volume of crystal/beam intersection. Shot-to-shot variation in the limiting resolution has been previously noted for microcrystal populations of two proteins: PSII and thermolysin (Kern *et al.*, 2013 ▶, 2014 ▶; Hattne *et al.*, 2014 ▶). These reports, using data processed with *cctbx.xfel*, were based on the examination of Wilson plots (integrated Bragg spot intensity *versus* diffraction angle bin), to identify the limiting resolution where average intensity falls below the average counting-statistics noise. This analysis, however, does not convey whether the resolution limits are determined by actual falloff of the recorded spot intensities or by artifacts produced by the integration algorithm.

**Figure 3:**
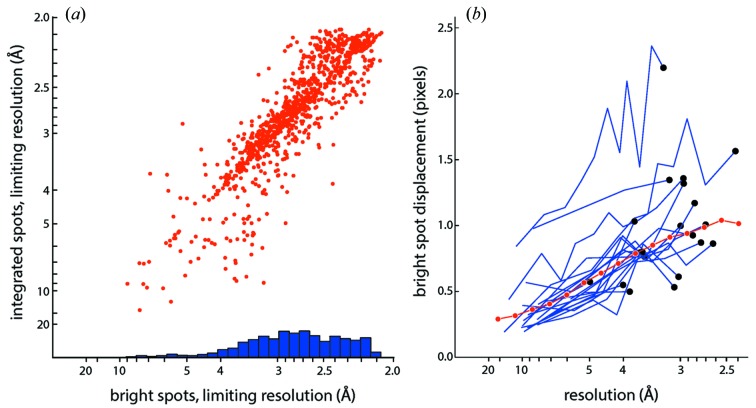
Resolution limits and positional accuracy of the thermolysin integration model. (*a*) Limiting resolution for 1000 randomly selected shots from runs 21–27 of the L498 experiment, collected at a sample-to-detector distance of 171.0 mm, and thus restricted to 2.6 Å at the detector edge, and 2.05 Å in the detector corners. Data for the strongest-diffracting samples are therefore limited by a sharp cutoff due to detector geometry rather than the intrinsic sample diffraction. Horizontal axis: limits based on bright spots picked by a spotfinding algorithm (Zhang *et al.*, 2006 ▶); blue bars represent a histogram of resolution limits determined with ‘method 2’ from that paper. Vertical axis: limits based on a Wilson plot of the integrated intensities. (*b*) Displacement (in pixels) between Bragg spot positions predicted by the lattice model used for integration, and the center of mass positions actually measured for bright spotfinder-picked spots. Blue traces: displacement for 20 randomly selected shots, with bright spots from each shot grouped into resolution bins; black dots identify the highest-resolution bin for each individual shot. Red curve: aggregate displacement over the 1000 images analyzed in panel (*a*).

Fig. 3(*a*) ▶ confirms that the resolution limit variation is indeed intrinsic to the recorded data. The horizontal axis (scatter plot and histogram) reports the distribution of resolution limits judged by a spotpicking algorithm (Zhang *et al.*, 2006 ▶). After removal of untrusted pixels and subtraction of local background, the signal is judged by whether the intensity exceeds local variance by a given threshold; the resulting population of Bragg spot candidates is then plotted as a function of diffraction angle and a uniform cutoff criterion applied over the whole data set. Resolution cutoffs determined this way are therefore independent of all the ensuing data processing details such as indexing (discovery of basis vector candidates), choice of basis vectors to form the unit cell, model refinement, application of symmetry constraints, and choice of algorithms for spot prediction and signal integration.

However, once the integrated intensities are analyzed with a Wilson plot, the resolution cutoffs of the integrated data [Fig. 3(*a*) ▶, vertical axis] are well correlated with those determined simply on the basis of spotpicking [correlation coefficient 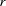 = 89% for Fig. 3(*a*) ▶], ruling out any distortion arising from data processing. Indeed, the lattice model used for data integration can be used to push beyond the limits of the spotfinder to some extent: for two-thirds of the images plotted (Fig. 3*a* ▶), the *cctbx.xfel* integration limit is above the diagonal, and therefore the model is finding signal that is missed by straightforward spotpicking.

Given this successful result, why not simply ignore the resolution cutoffs altogether, use the lattice model to predict spot positions out to the corner of the detector, and thereby take full advantage of the weak measurements when ultimately the duplicate measurements are merged over the whole data set to produce structure factors? Indeed, it is widely recognized (*e.g.* Weiss, 2001 ▶) that high-quality reduced data can be obtained by merging numerous multiplicitous measurements. The argument against this proposition is that it supposes that the error model for the weak high-resolution spots is well characterized and suitably random, which is a requirement for merging data (Borek *et al.*, 2003 ▶). The following sections (§§3.2–3.3) show that there are large non-random systematic uncertainties in the model. Moreover, while the spotpicking Bragg candidates offer a built-in validation of the model, the uncertainties are poorly characterized at the highest resolutions that are beyond the spotpicking limit.

### 3.2. The positional accuracy of the model is resolution-dependent   

The CSPAD imaging detector at LCLS (Hart *et al.*, 2012 ▶) is designed to fulfil stringent requirements: signal is integrated over a 50 fs X-ray pulse, readout is performed at the pulse repetition rate of 120 Hz, and operation is in vacuum. A large detection area is achieved by tiling 32 rectangular silicon sensors; however, this geometrical arrangement also creates the problem of knowing the sensors’ relative positions (metrology) to subpixel accuracy. As reported earlier (Hattne *et al.*, 2014 ▶), *cctbx.xfel* can determine or validate the tile displacements to within 0.1 pixel. It compares the positions of Bragg spots observed by the spotfinder with those predicted by the lattice model, and performs iterative non-linear least-squares parameter refinement over tile positions and lattice model parameters. Once the tile positions (and rotations) have been corrected, one can investigate the residual displacement errors of the bright Bragg spots (Fig. 3*b* ▶).

Fig. 3(*b*) ▶ indicates that the positional error of the model increases at higher resolutions; this is evident both for individual images (blue traces), and for aggregate positional errors over the whole dataset (red curve). While positional uncertainty is quite manageable at 10 Å *d*-spacings (0.3 pixels), it becomes problematic (1.0 pixel) at 2.7 Å. Several factors may combine to cause this effect. Firstly, there is a positional error, potentially 1 pixel or greater, due to the assumption of monochromaticity. In reality the X-ray pulses at LCLS have a stochastic spectrum with ∼0.5% bandpass (Emma *et al.*, 2010 ▶). Ideally the model could be augmented with a spot prediction algorithm that determines the wavelength range satisfying Bragg’s law separately for each reflection (Hattne *et al.*, 2014 ▶), thus taking the bandpass into account when predicting the 2θ diffraction angle. Secondly, the thickness of the silicon sensor (0.5 mm for the CSPAD) introduces a differential parallax effect, which again is potentially correctable (Hülsen *et al.*, 2005 ▶). These phenomena affect spots’ radial positions, and indeed we observe that the radial displacement is the largest component of the positional error (data not shown).

Regardless of the cause of the positional displacement shown in Fig. 3(*b*) ▶, values exceeding 1 pixel could significantly degrade the intensities, considering that a typical spot area is 5 square pixels (Hattne *et al.*, 2014 ▶), and in view of *cctbx.xfel*’s practice of constructing tightly conforming integration masks based on nearby bright spots. Rather than explicitly determining the uncertainty for each modeled spot at high resolution, *cctbx.xfel* currently takes the easier route of using the falloff of the Wilson plot as a proxy for uncertainty, and simply cutting off the integrated intensities past the apparent resolution limit. Other approaches to downweighting outlier data may be possible; for example, one of the 20 lattices plotted in Fig. 3(*b*) ▶ has positional displacements exceeding 2 pixels, which should probably disqualify it from the subsequent data merging process. Filtering individual lattices based on positional displacement rather than *I*/σ(*I*) falloff might offer a way to preserve weak high-resolution signal in the final merged intensities.

### 3.3. Resolution-dependent model quality, due to misorientation, affects map features   

An inherent concern with still shots is that the orientation of the crystal is not uniquely determined by measuring the Bragg spot positions. Only one of the three rotational degrees of freedom is directly coupled to spot positions, namely the rotation *R*_*z*_ around the axis parallel to the beam. The other two rotations (*R*_*xy*_) move reciprocal lattice points in and out of the reflecting condition, but do not change the direction of the diffracted rays. It has been possible to improve the outcome by placing an additional restraint on the refinement of the orientational model. Specifically, one can rotate the model lattice, while minimizing the deviations between the observed reciprocal lattice points and the Ewald sphere, with deviations being expressed either as reciprocal space distances (Kabsch, 2014 ▶) or rotational angles (Sauter *et al.*, 2014 ▶).

**Figure 4:**
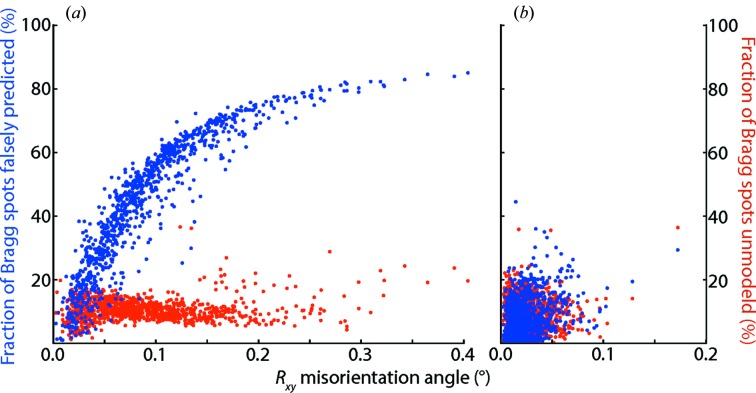
Bragg spot predictions are more accurate when the orientational model is refined against Ewald sphere distance. Two protocols are evaluated: (*a*) refinement of indexed spots against observed positions only, and (*b*) also refining the model against the angular deviation of the reciprocal lattice point from the Ewald sphere, corresponding to protocols 4 and 6 of Sauter *et al.* (2014 ▶), respectively. Plots represent a random sampling of processing results for simulated PSI data, in which the modeled orientation can be compared against the known true orientation from the simulation. Horizontal axis: residual misorientation angle *R*_*xy*_ after removal of the small misorientation *R*_*z*_ along the axis parallel to the beam direction (r.m.s. *R_z_* misorientation is 0.017° for both panels). Vertical axis: fraction of Bragg spots predicted by the model but not present in the simulated data (blue), and fraction of Bragg spots in the simulation that are not modeled (red).

The effect of these restraints can be directly gauged by considering simulated diffraction data. Fig. 4 ▶ shows that, for 1000 simulated 3.3 Å PSI diffraction patterns in random orientations, lattice models refined against spot positions alone have residual *R*_*xy*_ misorientations up to 0.3° (Fig. 4*a* ▶); while applying the angular restraint brings most *R*_*xy*_ mis­orientations to below 0.05° (Fig. 4*b* ▶). A misoriented lattice model can have a dramatic effect on spot predictions (Fig. 4*a* ▶). Improperly oriented model lattices place the observed reciprocal lattice points far away from the Ewald sphere, thus the mosaicity parameter must be adjusted upward so that the predicted spot pattern can cover all the observations. As illustrated in Fig. 4(*a*) ▶, this has the unwanted effect of creating false predictions for numerous spots that are not actually recorded in the image. Furthermore, previous work (Sauter *et al.*, 2014 ▶) with the PSI simulation shows that the fraction of spots predicted falsely increases with resolution. In parallel, if the experimental thermolysin data are processed with a protocol that omits the angular restraint and thus is believed to allow numerous false spot predictions, the ability to distinguish the Zn^2+^ anomalous difference Fourier peak is markedly decreased (Table 1 ▶, compare protocols 4 and 6). All these results provide further argument for cutting off integrated intensities at the resolution suggested by the Wilson plot, thereby guarding against the chance that any given measurement is errantly modeled due to lattice misorientation.

### 3.4. Direct test of the *I*/σ(*I*) cutoff   

The preceding two sections raise cautions about the systematic errors present in high resolution data. Accordingly, the program *cctbx.xfel* has been implemented with the option of applying a separate resolution cutoff to individual lattices, reasoning that, for the highest-resolution bins where *I*/σ(*I*) falls below a particular threshold, the data integration model has probably diverged too much for the intensities to be useful (Hattne *et al.*, 2014 ▶). However, as recent literature has highlighted the pitfalls of discarding data (Karplus & Diederichs, 2012 ▶; Diederichs & Karplus, 2013 ▶), Table 1 ▶ presents a direct comparison between thermolysin data processed with an *I*/σ(*I*)-dependent cutoff (protocols 6 and 7POST) and data processed with a fixed cutoff of 2.2 Å (6F and 7F,POST). As expected, the inclusion of more weak high-resolution data dramatically increases the average multiplicity of observations, as well as increasing the fraction of negative observations due to the poor quality of the high resolution models. Notably, however, the inclusion of more data also increases the height of the Zn^2+^ anomalous difference Fourier peak, suggesting that there is value in preserving the high resolution information. As noted in §3.2, it is worth developing alternative methods that would include more data, but yet still account for the known systematic errors such as positional displacement.

### 3.5. Modeling the partiality   

Even with utmost care given to choose data based on the significance level of the signal, a large inherent uncertainty remains with all still-shot data, due to the partial nature of the recorded intensities. This uncertainty is not present for rotation photography, where well established methods exist (Rossmann *et al.*, 1979 ▶; Winkler *et al.*, 1979 ▶) to quantify the spot partiality based on the volume of the reciprocal lattice point (rlpQ) swept up by the Ewald sphere due to rotation. However, this is not a useful measure for still shots where, in the absence of rotation, the swept-up volume due to rotation is always zero.

Two factors are directly relevant when considering spot partiality on still shots. First, due to crystal imperfection (Nave, 1998 ▶; Bellamy *et al.*, 2000 ▶; Helliwell, 2005 ▶), the reciprocal lattice point itself is spread out into a finite volume, therefore it has a finite intersection area with the Ewald sphere, even though the swept-up volume is zero. Secondly, due to the dispersion and divergence of the beam, one must consider a family of Ewald spheres of different radii (to account for dispersion; Hattne *et al.*, 2014 ▶) and radius vector direction (to account for divergence). This Ewald sphere degeneracy does sweep out a volume of the reciprocal lattice point, as has been discussed (White, 2014 ▶). In this paper, the focus is exclusively on the component of partiality due to crystal imperfection, as it seems a reasonable starting point. Many still datasets are acquired on endstations with negligible divergence, such as the LCLS/CXI 1 µm focus. While beam dispersion has been large (∼0.5%) for many XFEL datasets (Emma *et al.*, 2010 ▶), it is also possible to acquire stills at synchrotron sources where the energy bandpass 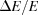 is potentially less then 10^−4^, and it is now possible to create seeded XFEL beams with similarly narrow bandpasses (Amann *et al.*, 2012 ▶).

Therefore, a correction for partiality based on a monochromatic zero-divergence model is described in equation (6). Partiality is related to the finite width of the reciprocal lattice spot, due to the underlying mosaic disorder in the crystal that is modeled by two parameters: an effective mosaic block size 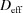 and an effective full-width mosaic angular spread 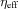. Intensity measurements are corrected for partiality in combination with scaling and postrefinement [equations (8) and (9)]. Despite the simple assumption of monochromaticity, this treatment notably improves the XFEL thermolysin data, which were collected with a non-monochromatic source (Table 1 ▶, compare protocols 6 and 7POST). The multiplicity of observation decreases, due to many reciprocal lattice points being classified as lying too far from the Ewald sphere, thus discarding a set of measurements that have no signal. The crystallographic *R*-factors improve, and the significance level of the anomalous difference Fourier peak for the Zn increases from 5.8σ to 7.4σ. These effects depend on performing postrefinement [equation (9)] to determine the optimal crystal orientation for calculating partiality; no improvement is observed unless the partiality correction is combined with postrefinement (data not shown).

**Figure 5:**
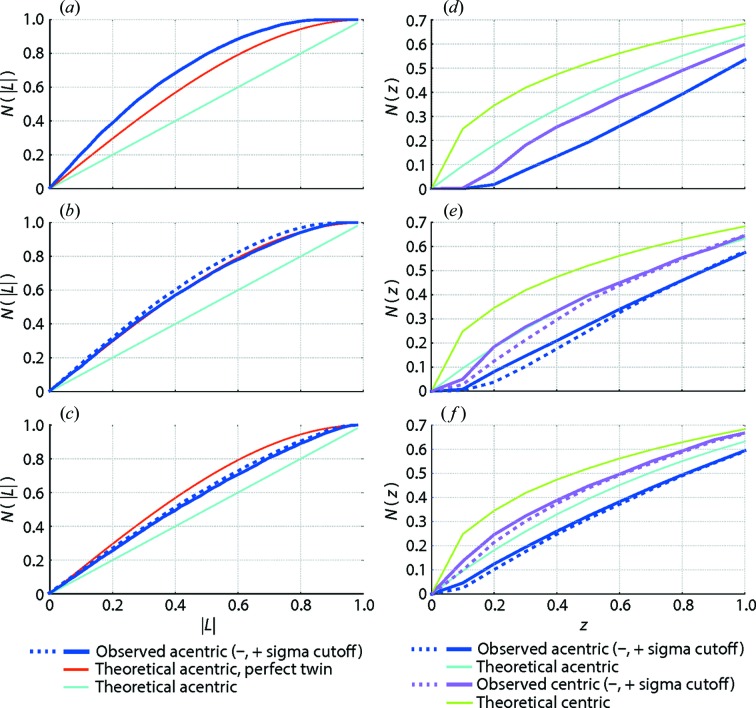
Data quality statistics for the merged structure factor intensities from thermolysin. *(a*, *b*, *c*) Cumulative distribution function *N*(*L*) of the local statistic: *L* = 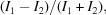 where 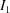 and 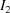 are unrelated intensities (Padilla & Yeates, 2003 ▶). (*d*, *e*, *f*) Cumulative distribution function *N*(*z*), where *z* = *I*/〈*I*〉. Identical data were processed with the protocols listed in Table 1 ▶: (*a*, *d*) protocol 4, lattice model is not restrained against proximity to the Ewald sphere; (*b*, *e*) protocols 6 and 6F, proximity restraints are applied, with and without a separate resolution cutoff for each lattice; and (*c*, *f*) protocols 7POST and 7F,POST, which are the same as protocols 6 and 6F except that crystal orientation is postrefined to maximize agreement with a set of reference intensities as described in the text. Agreement between the merged intensities (thick lines) and the theoretical distribution (thin lines) demonstrates that such statistics offer useful metrics for evaluating different processing protocols, with the postrefined model giving the best agreement with theoretical expectation.

Statistics indicating the quality of the merged structure factors (Padilla & Yeates, 2003 ▶) also show that the partiality correction (with postrefinement) alters the intensity distribution so as to conform better with theoretical expectation (Table 1 ▶ and Fig. 5 ▶). Synchrotron datasets have long been judged by their structure factor intensity distributions (Wilson, 1949 ▶; French & Wilson, 1978 ▶; Stein, 2007 ▶). It would be useful if such metrics could also be applied to judge the quality of XFEL data. However, the present comparison shows that distributions of the *L* and *Z* statistics (defined in Fig. 5 ▶) are highly dependent on the data processing procedures, and that, while accounting for partiality helps, the optimal protocol has not yet been achieved. One straightforward avenue for improvement would be to incorporate known spectral dispersion information into the partiality calculation. XFEL pulses, in particular the self-amplified stimulated emission pulses (SASE) in ordinary use, have complex and stochastic spectra, but it has been possible to measure these spectra on a shot-by-shot basis (Zhu *et al.*, 2012 ▶). For future datasets where the incident spectra 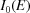 are routinely available, one could perform a weighted summation over the entire bandpass to obtain the polychromatic partiality,
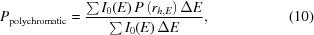
where the summations are performed over all energy increments 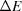 within the measured spectrum, and the functional dependence of 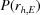 is explicitly stated to emphasize that the Ewald-sphere distances 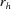 are dependent on energy. Spectral measurements are not available for the thermolysin data presented here; however, other datasets that are linked to spectral information are under investigation.

## 4. Conclusions   

To the knowledge of the author, this is the first literature presentation of experimentally measured XFEL still-shot diffraction data that are explicitly corrected for partiality (albeit with the simplified assumption of monochromaticity), and modeled with a lattice that is oriented by postrefinement. Equation (6), the expression for still-shot partiality, is similar to equation (40) in a recent paper from Kabsch (2014 ▶), in that both rest on the assumption of monochromaticity. However, the Kabsch paper does not include the effect of mosaic block size [equation (2)], which makes a resolution-independent contribution to the size of reciprocal lattice points, necessary for optimal modeling of still data (Sauter *et al.*, 2014 ▶) if the block size is small. The equation (6) approach differs substantially from that used by White (2014 ▶), as that paper defines partiality in terms of the fractional immersion of a reciprocal lattice point between two Ewald spheres of different wavelengths, representing the high- and low-energy limits of the XFEL spectrum.

While no attempt is made here to comparatively evaluate these three partiality and postrefinement methodologies, it is clear that, as a general principle, algorithm choices must rely on objective metrics that measure the quality of the result. Examples of data processing quality metrics include the r.m.s. displacement between observed and modeled Bragg spot positions (and its resolution dependence), statistics that rely on the moments of the intensity distribution (Stein, 2007 ▶), local *L*-statistics (Padilla & Yeates, 2003 ▶), crystallographic *R*-factors, and the height of anomalous difference Fourier peaks for metal sites.

Thermolysin is an informative case for testing the potential of still-shot crystallography. It is possible to phase the structure with synchrotron data using SAD phasing, from the single Zn metal site (Ferrer *et al.*, 2013 ▶). However, the best XFEL thermolysin data (giving an 18σ anomalous difference Fourier peak out to 1.8 Å resolution) falls short of the phasing power needed for a SAD structure solution (Kern *et al.*, 2014 ▶). Only a single SAD-phased XFEL structure has been published (of lysozyme; Barends *et al.*, 2013*b* ▶), yet the usefulness of XFEL techniques may depend on whether they can be utilized generally to solve new macromolecular structures, and gain high-resolution information on systems that would otherwise be damaged at synchrotron sources. Data processing strategies that help correct specific issues such as partial measurements and the heterogeneous distribution of resolution limits will hopefully lead to more favorable structural outcomes.

## 5. Software availability   

The partiality correction and postrefinement procedures described here are incorporated into *cctbx.xfel* (http://cci.lbl.gov/xfel) and are available as a command line option in the *cxi.merge* program component.

## Supplementary Material

Figure S1: Likelihood-weighted electron density maps. DOI: 10.1107/S1600577514028203/xh5046sup1.pdf

## Figures and Tables

**Table 1:** Processing outcome on XFEL still shots from thermolysin

	Protocol number				
	4	6	6F	7POST	7F,POST
Protocol choice					
Model restraints	Spot positions only	Spot positions + angular deviations	Spot positions + angular deviations	Spot positions + angular deviations	Spot positions + angular deviations
Postrefinement and partiality correction	No	No	No	Yes	Yes
Each-lattice resolution bin cutoff [*I*/(*I)*]	0.5	0.5	None	0.5	None
Indexing results†					
# Total hits with >15 Bragg spots	14041	14041	14041	14041	14041
# Integrated and merged lattices	12097	12550	13756	12551	13733
Model accuracy					
Half-width mosaicity	0.292	0.168	0.213	0.168	0.213
Mosaic block size	4320	4220	4370	4220	4370
Integrated data results					
Individual image CC	32.0%	40.2%	40.1%	40.2%	40.1%
No. of measurements 512.2	6605566	5036076	11905131	4290566	9915864
Positive measurements 512.2	4297065	3626262	7249271	3187835	6201772
Negative measurements	35%	28%	39%	26%	37%
Structure factor merging					
Merging resolution range ()	512.2 (2.282.2)	512.2 (2.282.2)	512.2 (2.282.2)	512.2 (2.282.2)	512.2 (2.282.2)
Unique Miller indices	17198 (1405)	17297 (1488)	17513 (1700)	17227 (1425)	17513 (1700)
Multiplicity of observation	250 (3.0)	210 (3.6)	414 (53)	185 (3.2)	354 (44)
Completeness	98.2% (82.7%)	98.8% (87.6%)	100% (100%)	98.4% (83.9%)	100% (100%)
*I*/(*I*)	36.1 (2.3)	56.7 (3.2)	55.9 (4.2)	74.9 (3.5)	72.7 (4.0)
CC_1/2_ correlation of semi-datasets	72.2% (4.1%)	87.2% (42.7%)	92.1% (14.6%)	90.2% (34.0%)	92.8% (16.0%)
*R*_1/2_ intensity agreement of semi-datasets	33.9% (95.2%)	32.0% (89.7%)	26.7% (69.6%)	29.3% (89.7%)	26.7% (78.0%)
CC_iso_ *versus* 4ow3 (based on intensities)	86.8% (18.1%)	94.7% (40.0%)	95.1% (23.3%)	94.8% (42.1%)	95.2% (30.1%)
*R*_iso_ *versus* 4ow3 (based on intensities)	23.6% (79.0%)	18.0% (73.8%)	17.7% (63.8%)	23.4% (76.1%)	22.5% (69.3%)
Structure factor quality tests					
Wilson *B*-factor (^2^)	12.2	17.2	18.3	17.7	20.6
*I* ^2^/*I* ^2^ (theoretical 2.0)	1.293	1.518	1.471	1.697	1.628
|*L*| (acentric theoretical = 0.5)	0.302	0.376	0.366	0.425	0.412
*L* ^2^ (acentric theoretical = 0.333)	0.137	0.202	0.193	0.252	0.238
*N*(*Z*) maximum deviation (acentric)	0.201	0.121	0.133	0.071	0.082
*N*(*Z*) maximum deviation (centric)	0.271	0.198	0.213	0.112	0.147
Quality of refined structure					
Refinement resolution range ()	512.2 (2.342.2)	512.2 (2.342.2)	512.2 (2.342.2)	512.2 (2.342.2)	512.2 (2.342.2)
*R*_work_	24.5% (35.2%)	20.8% (33.5%)	21.2% (32.0%)	20.6% (36.3%)	19.5% (33.0%)
*R*_free_	29.6% (39.9%)	26.3% (44.0%)	26.0% (39.0%)	24.1% (45.8%)	24.3% (42.0%)
Zn^2+^ anomalous-difference peak height	2.9	5.8	7.2	7.4	8.7
Molprobity clashscore (Chen *et al.*, 2010 ▶)	8.41	2.16	3.23	1.08	0.86
Protein atom *B*-factors (^2^)	15.6	18.0	20.4	19.1	21.3
Solvent atom *B*-factors (^2^)	23.3	28.8	29.7	27.9	30.5
Number of autobuilt water molecules	311	295	248	236	232
Overall/local map C.C. to 2tlx model	77.0%/81.3%	81.3%/84.4%	82.0%/85.0%	82.5%/85.2%	83.2%/85.7%
Automated model building after MR-SAD					
No. of mainchain/sidechain (of total 316)	310/299	309/309	309/309	312/305	312/306
*R*_work_/*R*_free_	24.0%/28.8%	23.7%/28.2%	22.6%/26.2%	23.0%/26.5%	22.6%/26.2%

† For the thermolysin data analysis, candidate Bragg spots were chosen with a minimum spot area of 2 square pixels.

## Footnotes

1 The earlier paper (Sauter *et al.*, 2014 ▶) treats  as a rotational spread about the origin *O* of reciprocal space, causing rlpQ to be shaped as a spherical cap of radius 1/*d* and maximum half-angle . Here, in contrast, equation (3) implies that rlpQ is shaped like a ball, not a spherical cap. This is chosen only to simplify the derivation of partiality, not to indicate a preference for a particular physical model (Nave, 1998 ▶; Juers *et al.*, 2007 ▶) to describe the crystal.

2 Scaling with a set of reference intensities has been used in more traditional crystallographic settings to extract weak signal from long wavelength anomalous diffraction experiments (Mueller-Dieckmann *et al.*, 2004 ▶). Moreover, it can be shown with XFEL data that a scaling reference introduces no intensity bias, by scaling XFEL lysozyme measurements (CXIDB accession ID 17) against an isomorphous lysozyme structure containing the alanine truncation mutant E35A (PDB entry 3ok0). After molecular replacement (with the correct structure 4et8 used as the search model) followed by refinement, the likelihood-weighted electron density map shows perfectly normal signal for glutamic acid 35, proving that the false scaling model does not distort the information content of the experimental intensities. Furthermore, if the 3ok0 structure is used for phasing instead of 4et8, one sees positive difference density for the glutamate side chain at 4 standard deviations, indicating that the intensities contain sufficient signal to overcome the phase bias introduced by the incorrect 3ok0 phasing model.

3 Supporting information for this paper is available from the IUCr electronic archives (Reference: XH5046).
