# Supplementary material for: XFEL diffraction: developing processing methods to optimize data quality
Source: J Synchrotron Radiat. 2015 Jan 29;22(Pt 2):239–48. doi: 10.1107/S1600577514028203 (PMC4344359; doi:10.1107/S1600577514028203)
Supplement: Supplementary file 1 [file s-22-00239-sup1.pdf]

JOURNAL OF SYNCHROTRON RADIATION

Volume 22 (2015)

Supporting information for article:

XFEL diffraction: developing processing methods to optimize data quality

Nicholas K. Sauter

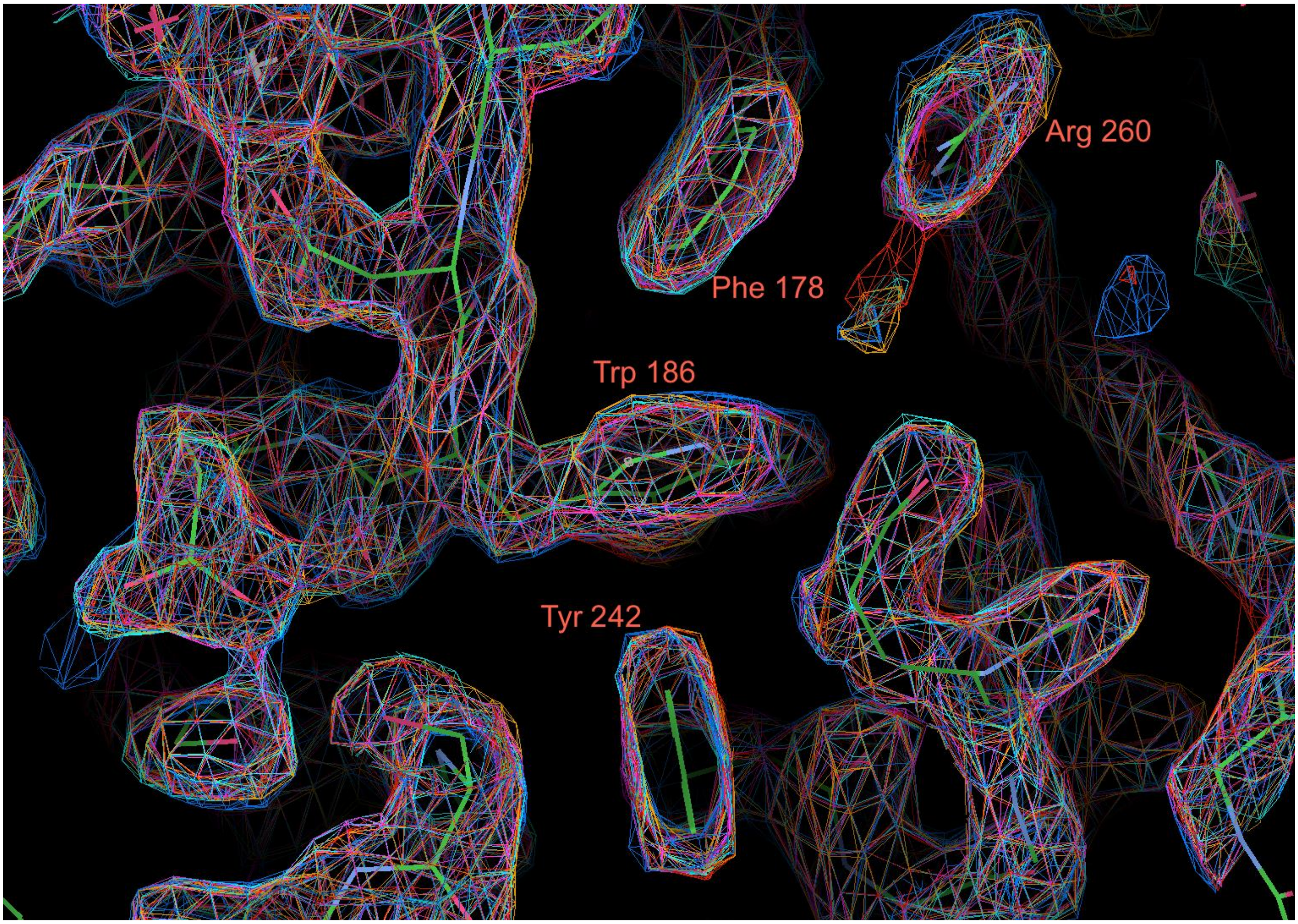

Figure S1 Figure S1. Likelihood-weighted electron density maps with coefficients $2mF_o$–DF_c. The thermolysin molecular model is refined against data processed with protocol 7POST (Table 1), while electron density maps contoured at +1.2σ are illustrated for all protocols: 4 (blue), 6 (cyan), 6F (orange), 7POST (magenta), and 7F,POST (red). Contours surrounding the amino acid sidechains are in approximate agreement across all data-processing protocols, while the details differ in some locations, including those defining the surrounding solvent atoms.
